# Treatment Strategies and Outcome of the Exstrophy–Epispadias Complex in Germany: Data From the German CURE-Net

**DOI:** 10.3389/fped.2020.00174

**Published:** 2020-05-19

**Authors:** Anne-Karoline Ebert, Nadine Zwink, Heiko M. Reutter, Ekkehart Jenetzky, Raimund Stein, Alice C. Hölscher, Martin Lacher, Caroline Fortmann, Florian Obermayr, Margit Fisch, Kiarasch Mortazawi, Eberhard Schmiedeke, Martin Promm, Karin Hirsch, Frank-Mattias Schäfer, Wolfgang H. Rösch

**Affiliations:** ^1^Department of Pediatric Urology, University Hospital for Urology and Pediatric Urology, University Medical Center Ulm, Ulm, Germany; ^2^Department of Child and Adolescent Psychiatry, University Medical Center of the Johannes Gutenberg University Mainz, Mainz, Germany; ^3^Department of Neonatology and Pediatric Intensive Care, Children's Hospital, University of Bonn, Bonn, Germany; ^4^Institute of Human Genetics, University Hospital Bonn, Bonn, Germany; ^5^Child Center Maulbronn GmbH, Hospital for Pediatric Neurology and Social Pediatrics, Maulbronn, Germany; ^6^Department of Pediatric and Adolescent Urology, University Medical Center Mannheim, Mannheim, Germany; ^7^Department of Pediatric Surgery and Pediatric Urology, Children's Hospital Amsterdamer Straße Köln, Köln, Germany; ^8^Department of Pediatric Surgery, University Hospital Leipzig, Leipzig, Germany; ^9^Center of Pediatric Surgery Hannover, Hannover Medical School and Bult Children's Hospital, Hannover, Germany; ^10^Department of Pediatric Surgery and Pediatric Urology, University Hospital for Child and Adolescent Medicine Tübingen, Tübingen, Germany; ^11^Department of Urology and Pediatric Urology, University Medical Center Hamburg-Eppendorf, Hamburg, Germany; ^12^Department of Pediatric Surgery, Klinik für Kinderchirurgie, Städtisches Klinikum Karlsruhe, Karlsruhe, Germany; ^13^Department of Pediatric Surgery and Pediatric Urology, Center for Child and Youth Health, Klinikum Bremen-Mitte, Bremen, Germany; ^14^Department of Pediatric Urology, Clinic St. Hedwig, University Medical Center Regensburg, Regensburg, Germany; ^15^Department of Pediatric Urology, University Hospital Erlangen, Erlangen, Germany; ^16^Pediatric Surgery and Pediatric Urology, Cnopfsche Children's Hospital, Nürnberg, Germany

**Keywords:** exstrophy–epispadias complex (EEC), operative outcome, outcome assessment, treatment strategies, staged approach, single-stage approach, post-operative complications

## Abstract

**Introduction:** To evaluate the impact of reconstructive strategies and post-operative management on short- and long-term surgical outcome and complications of classical bladder exstrophy (CBE) patients' comprehensive data of the multicenter German-wide Network for Congenital Uro-Rectal malformations (CURE-Net) were analyzed.

**Methods:** Descriptive analyses were performed between 34 prospectively collected CBE patients born since 2009, median 3 months old [interquartile range (IQR), 2–4 months], and 113 cross-sectional patients, median 12 years old (IQR, 6–21 years).

**Results:** The majority of included individuals were males (67%). Sixty-eight percent of the prospectively observed and 53% of the cross-sectional patients were reconstructed using a staged approach (*p* = 0.17). Although prospectively observed patients were operated on at a younger age, the post-operative management did not significantly change in the years before and after 2009. Solely, in prospectively observed patients, peridural catheters were used significantly more often (*p* = 0.017). Blood transfusions were significantly more frequent in males (*p* = 0.002). Only half of all CBE individuals underwent inguinal hernia repair. Cross-sectional patients after single-stage reconstructions showed more direct post-operative complications such as upper urinary tract dilatations (*p* = 0.0021) or urinary tract infections (*p* = 0.023), but not more frequent renal function impairment compared to patients after the staged approach (*p* = 0.42). Continence outcomes were not significantly different between the concepts (*p* = 0.51). Self-reported continence data showed that the majority of the included CBE patients was intermittent or continuous incontinent. Furthermore, subsequent consecutive augmentations and catheterizable stomata did not significantly differ between the two operative approaches. Urinary diversions were only reported after the staged concept.

**Conclusions:** In this German multicenter study, a trend toward the staged concept was observed. While single-stage approaches tended to have initially more complications such as renal dilatation or urinary tract infections, additional surgery such as augmentations and stomata appeared to be similar after staged and single-stage reconstructions in the long term.

## Introduction

Worldwide, enormous efforts have been made to improve operative techniques of the exstrophy–epispadias complex (EEC) (1–7). For a considerable long period, however, major reconstructive continence concepts in classical bladder exstrophy (CBE) remained unchanged, although long-term results in regard of bladder function and achievable continence vary significantly. There is still no consensus about the best operative strategy in CBE in respect of future bladder and upper tract outcome or the need for consecutive operations. This was the cornerstone for initiation of the multicenter German-wide population-based Network for Congenital Uro-REctal malformations (CURE-Net) with its comprehensive national data collection on EEC patients. The aim of this current study was to analyze CBE treatment practice over a long-term period with a focus on operative treatment strategies, their early and late post-operative complications, and consecutive operations to achieve continence. This study is the first to compare a prospectively observed with a cross-sectional cohort of CBE patients in a nationwide survey.

## Patients and Methods

### Study Population

In CURE-Net, individuals with EEC are identified and recruited through participating departments of pediatric urology and pediatric surgery throughout Germany and the two German self-help organizations Blasenekstrophie/Epispadie e.V. and Kloakenekstrophie e.V. Since 2009, the centralized database comprises clinical data of a prospectively observed cohort (infant baby, ≤ 1 year old at the time of data acquisition) and a cross-sectional cohort (>1 year at the time of data acquisition) of EEC individuals throughout Germany. Data of the exstrophy group since 2009 were collected prospectively, starting at the time of reconstruction through the treating physician and still ongoing by recontacting EEC patients over time. Patients older than 18 years and parents of affected minors were personally interviewed by a physician using a standardized questionnaire. Additionally, cross-sectional patients' data were also retrieved from hospital letters and charts if available. Written informed consent was obtained from all subjects or their legal guardians in case of minors. This study was approved by each participating center's Institutional Ethics Committee (e.g., University of Regensburg No. 09/053, University of Ulm No. 425/13).

### Structure of Data Analysis

From May 2009 to December 2016, 59 prospectively observed and 178 cross-sectional EEC patients were enrolled in the complete CURE-Net cohort. Current analysis focused exclusively on CBE patients, including 34 prospectively observed infant babies from 2009 onward [median, 3 months; interquartile range (IQR), 2–4 months; 10 females (29%), 24 males (71%); male-to-female ratio 2.4:1] and 113 cross-sectional patients [median, 12 years (IQR, 6–21 years); 39 females (35%), 74 males (65%); male-to-female ratio 1.9:1]. Further 19 cross-sectional CBE patients (3 females, 16 males) were excluded because of operative technique (primary urinary diversion).

Operative techniques were differentiated between a single-stage approach with bladder closure obligatory including a bladder neck reconstruction, pelvic ring adaptation, genital reconstruction, and optional with ureterocystoneostomy (in Germany mostly “Erlangen technique” (8) or CPRE) and a staged approach with postnatal bladder closure alone and further procedures to reconstruct the bladder neck and genitalia at a later time in life. In both approaches, pelvic ring adaptation technique with a simple traction bandage and intraoperative absorbable polydioxanone sutures of the pubic bones (9) was used for most exstrophy patients. After immobilization with a mummy wrap for about 10 days, a substantial connective tissue bridge is formed (9).

Expert consensus defined perioperative management strategies as parts of a standardized perioperative treatment recommendation (Table 1) and perioperative and post-operative complications (Tables 1, 2). Continence definition was adapted to the International Children's Continence Society (ICCS) terminology, referring to continuous urinary continence, intermittent, and continuous urinary incontinence (10).

**Table 1:** Perioperative management and complications according to initial operative technique.

	**Prospectively observed patient group (*****n*** **=** **34)**			**Cross-sectional patient group (*****n*** **=** **113)**		
	**Staged approach (*n* = 23)**	**Single-stage approach (*n* = 11)**	***P*^Δ^**	**Staged approach (*n* = 60)**	**Single-stage approach (*n* = 53)**	***P*^Δ^**
**Postoperative intensive care observation**			1.0			0.29
Yes	21 (90%)	8 (73%)		51 (84%)	42 (80%)	
No	0	0		7 (13%)	2 (6%)	
Missing data	2 (7%)	3 (20%)		2 (3%)	9 (14%)	
**Peridural catheter (PDC)**			**0.009**			0.84
Yes	18 (64%)	2 (33%)		22 (39%)	20 (37%)	
No	3 (29%)	5 (40%)		30 (43%)	23 (41%)	
Missing data	2 (7%)	4 (27%)		8 (19%)	10 (22%)	
**Wound and tissue infection**			0.36			0.43
Yes	4 (13%)	3 (33%)		22 (32%)	11 (22%)	
No	17 (81%)	5 (47%)		32 (60%)	32 (58%)	
Missing data	2 (7%)	3 (20%)		6 (8%)	10 (20%)	
**Blood transfusion**			0.68			0.19
Yes	13 (45%)	4 (40%)		23 (32%)	13 (26%)	
No	8 (48%)	4 (40%)		31 (60%)	30 (49%)	
Missing data	2 (7%)	3 (20%)		6 (8%)	10 (25%)	
**Anticholinergic medication**			0.27			0.59
Yes	18 (71%)	5 (53%)		36 (64%)	24 (48%)	
No	2 (19%)	2 (20%)		9 (13%)	9 (17%)	
Missing data	3 (10%)	4 (27%)		15 (23%)	20 (35%)	
**Low-dose antibiotic prophylaxis**			1.0			1.0
Yes	21 (90%)	7 (73%)		55 (87%)	43 (78%)	
No	0	0		0	0	
Missing data	2 (%)	4 (27%)		5 (13%)	10 (22%)	
**Transurethral catheter in place (days)**			0.53^ΔΔ^			0.93^ΔΔ^
Median (IQR)	15.5 (14–19); Min 6, Max 28	20 (14–31); Min 0, Max 40		21 (19–29); Min 0, Max 80	21 (19–32); Min 0, Max 49	
**Discharge (days)**			0.11^ΔΔ^			0.17^ΔΔ^
Median (IQR)	21 (18–23); Min 10, Max 36	39.5 (22–46); Min 14, Max 57		27 (24–30); Min 17, Max 50	28.5 (23–42); Min 19, Max 150	

Δ *Calculated by the Fisher's exact test*.

ΔΔ *Calculated by the t-test*.

*IQR, interquartile range. Bold values are the significant ones p <0.05*.

**Table 2:** Postoperative complications after primary reconstruction.

**Complications**	**Prospectively observed patient group (*****n*** **=** **34)**			**Cross-sectional patient group (*****n*** **=** **113)**		
	**Staged approach (*n* = 23)**	**Single-stage approach (*n* = 11)**	***P*^Δ^**	**Staged approach (*n* = 60)**	**Single-stage approach (*n* = 53)**	***P*^**Δ**^**
**Urinary tract dilatation after reconstruction**			0.71			**0.0021**
Yes	11 (48%)	6 (55%)		11 (18%)	24 (45%)	
No	12 (52%)	5 (45%)		18 (30%)	19 (36%)	
Missing data	0	0		31 (52%)	10 (19%)	
**UTI after reconstruction**			**0.020**			**0.023**
Yes	7 (30%)	8 (73%)		26 (43%)	35 (66%)	
No	16 (70%)	2 (18%)		11 (18%)	10 (19%)	
Missing data	0	1 (9%)		23 (38%)	8 (15%)	
**Renal deterioration after reconstruction**			1.0			0.42
Yes	0	0		2 (3%)	4 (7%)	
No	21 (91%)	10 (91%)		28 (47%)	37 (70%)	
Missing data	2 (9%)	1 (9%)		30 (50%)	12 (23%)	
**Unilateral nephrectomy**			1.0			0.50
Yes	2 (9%)	0		2 (3%)	0	
No	21 (91%)	10 (91%)		58 (97%)	49 (93%)	
Missing data	0	1 (9%)		0	4 (7%)	
**Disturbed bladder function (diagnosed by urodynamic findings)**			1.0			**0.025**
Yes	0	0		1 (2%)	7 (13%)	
No	20 (87%)	9 (82%)		22 (37%)	27 (51%)	
Missing data	3 (13%)	2 (18%)		37 (62%)	19 (36%)	
Epidydimitis during follow-up^*^			1.0			0.10
Yes	0	0		8 (22%)	4 (11%)	
No	18 (100%)	5 (83%)		14 (38%)	23 (62%)	
Missing data	0	1 (17%)		15 (40%)	10 (27%)	

* *Only in male patients*.

Δ *Calculated by the Fisher's exact test*.

*UTI, urinary tract infection. Bold values are the significant ones p <0.05*.

### Statistical Analysis

Descriptive data of the study population, operative treatment strategies, and post-operative management are presented in absolute and relative frequencies. To assess possible differences between the prospectively observed and cross-sectional patient group, as well as between female and male patients, Fisher's exact test was used, and the *t*-test to calculate possible differences in symphysis width, length of transurethral catheter placement, and hospital stay. Statistical significance was defined by *p* <0.05. Analyses were performed by the statistics software SAS, version 9.4 (SAS Institute Inc., Cary, NC, USA).

## Results

### Operative Procedures

Operative approaches were performed almost in an equal proportion in the cross-sectional cohort (staged approach 53%, single-stage approach 47%). Twenty-three (68%) prospectively observed patients had a staged and 11 (32%) a single-stage procedure. Proportions of operative approaches between both cohorts were not statistically significant (*p* = 0.17). Mean age for primary reconstructions was 0.8 year in the cross-sectional and 0.3 year in the prospectively observed patient group. Subgroup analysis showed that single-stage approaches were done at a mean age of 0.4 year in the cross-sectional and of 0.6 year in the prospectively observed patient group. Interestingly, the average age of bladder closure within the staged approach was 0.2 year in the prospectively observed and 1.3 year in the cross-sectional cohort.

Inguinal hernia repair was reported in 44% of the prospectively observed and 52% of the cross-sectional patients. Thirty-seven percent of all included CBE patients did not undergo herniotomy during any primary or secondary procedure. In the prospectively observed group, primary hernia repair was performed almost equally in girls (40%) and boys (46%), with a bilateral repair in 87%. In the cross-sectional patient group, hernia repair was significantly more often done in males than in females (26% females, 66% males, *p* <0.0001; bilateral repair 76%). Redo surgery for recurrent hernias was necessary in five cross-sectional patients (4%). Symphysis diastasis with inguinal hernia compared to those with no inguinal hernia was significantly different neither in prospectively observed (*p* = 0.76) nor in cross-sectional patients (*p* = 0.06). Furthermore, no differences were observed between both groups (*p* = 0.23).

Symphysis adaptation was documented in 101 of 147 patients [69%; prospectively observed patients: *n* = 24 (71%), cross-sectional patients: *n* = 77 (68%)]. An osteotomy was performed in 7 of 113 cross-sectional patients [6%; 4 (4%) with staged approach, 3 (3%) with a single-stage approach] and in 3 of 34 prospectively observed patients [9%; 2 (6%) with staged approach, 1 (3%) with a single-stage approach]. The most common osteotomy type performed was posterior osteotomy; only two cross-sectional patients underwent an anterior osteotomy. From operative reports, median intraoperative symphysis width achieved at primary closure for CBE patients was 1 cm (*n* = 18; IQR, 0.5–1.2 cm) in the cross-sectional and 0 cm (*n* = 20; IQR, 0–1 cm) in the prospectively observed patient group.

### Post-Operative Management

When comparing perioperative management strategies as a part of a standardized perioperative treatment recommendation and possible perioperative complications, no significant differences were found between either the two operative techniques or both cohorts (Table 1). Only peridural catheters (PDCs) were inserted nearly twice as often in staged than in single-stage approaches in the prospectively observed group. When comparing both patient groups in general, a significantly higher frequency of PDCs was found in the prospectively observed than in the cross-sectional cohort (*p* = 0.017). Stratification for sex showed no differences between both patient groups regarding the incidence of post-operative intensive care observation (*p* = 0.49), PDC use (*p* = 0.55), wound infection (*p* = 1.0), and medication such as anticholinergic drugs (*p* = 1.0) or antibiotic prophylaxis (*p* = 0.25). In contrast, blood transfusions were found significantly more often in males (*p* = 0.002). Additionally, blood transfusions were predominant in males after a single-stage approach (*p* = 0.004) compared to a staged approach (*p* = 0.22). These cross-sectional patients with a blood transfusion after a single-stage approach did not have more frequently an osteotomy done, compared to those who had no blood transfusion (*p* = 0.95). There were no age differences in the prospectively observed cohort who needed a blood transfusion in regard of gender (*p* = 0.68) or operative approaches (*p* = 1.0).

Postoperatively, a transurethral catheter remained in place for a median of 16 days (IQR, 14–20 days) in the prospectively observed and for a median of 21 days (IQR, 19–35 days) in the cross-sectional patient group (*p* = 0.0013).

Although the proportion of the reconstruction methods varied between the prospectively observed and cross-sectional patients, there was no significant difference between the duration of catheter placement between the single-stage and the staged approach (prospectively observed cohort *p* = 0.53, cross-sectional cohort *p* = 0.93). Additionally, neither male nor female gender did influence the time of having a catheter in place (prospectively observed cohort *p* = 0.64, cross-sectional cohort *p* = 0.89). The prospectively observed patients stayed significantly shorter in hospital than did patients of the cross-sectional group [median, 21.5 days (IQR, 17–30 days) vs. median, 28 days (IQR, 24–35 days)] (*p* = 0.019). There were no differences in regard to either the mode of reconstruction (Table 1) or gender (prospectively observed group *p* = 0.83, cross-sectional group *p* = 0.28).

### Post-Operative Complications and Following Operations

In the cross-sectional cohort, urinary tract infection (UTI) and upper tract dilatation occurred significantly more often after single-stage than after staged approach (Table 2). In the prospectively observed group, UTIs were quite common after single-stage procedures and therefore significantly more often than after staged approaches (*p* = 0.02) (Table 2). However, subsequent impairment of renal function did not differ between the two groups (*p* = 0.42) or the prospectively observed and cross-sectional cohort. Renal function impairment was confirmed in two patients after staged approach by a renal scan with no further surgical therapy. Further two patients with a staged approach underwent nephrectomy. No patients with single-stage approach had a nephrectomy done; however, in four patients, renal function impairment was reported after sonography; all of them needed therapy such as ureterocystoneostomy or percutaneous kidney drainage. The significant frequency in disturbed bladder function must be treated with caution because of very small numbers and a high rate of missing data.

Augmentations with ileum (*p* = 0.67) and catheterizable stomata according the Mitrofanoff principle (Appendix *p* = 0.84, Monti *p* = 0.59) were necessary in about the same extent after the staged and the single-stage approach in cross-sectional patients (Table 3). Augmentations and catheterizable stomata were performed approximately at the same age after both reconstruction concepts (*p* = 0.57) [staged approach: median age, 9 years (IQR, 7–12 years); single-stage approach: median age, 9 years (IQR, 6–12 years)]. One patient received small intestinal submucosa (SIS) for augmentation with unfavorable outcome published elsewhere (11). Urinary diversions preferably by a rectosigmoid pouch or a conduit were reported as salvage procedures only after the staged approach (Table 3). A (redo) bladder neck plasty was performed in 15 patients (25%) with a staged concept as a regular reconstruction part at the median age of 6 years (IQR, 5–7 years). However, 26% of the single-stage reconstructed patients needed a true redo bladder neck plasty to improve continence, which was performed at school age [median, 6 years (IQR, 6–7 years)].

**Table 3:** Following operations to increase bladder capacity, improve bladder emptying, or gain urinary continence (bladder neck procedure) in the long-term follow-up.

**Following operations**	**Cross-sectional patient group (*****n*** **=** **113)**		
	**Staged approach (*n* = 60)**	**Single-stage approach (*n* = 53)**	***P*^Δ^**
**Bladder augmentation with**			
1. Ileum			0.67
Yes	15 (25%)	15 (28%)	
No	45 (75%)	36 (68%)	
Missing data	0	2 (4%)	
2. Colon			0.60
Yes	1 (2%)	2 (4%)	
No	59 (98%)	50 (94%)	
Missing data	0	1 (2%)	
3. Foreign material (such as SIS)			0.46
Yes	0	1 (2%)	
No	60 (100%)	51 (96%)	
Missing data	0	1 (2%)	
**Catherizable stoma according the Mitrofanoff principle**			
1. Appendix			0.84
Yes	20 (33%)	18 (34%)	
No	40 (67%)	32 (60%)	
Missing data	0	3 (6%)	
2. Monti			0.59
Yes	1 (2%)	2 (4%)	
No	59 (98%)	48 (90%)	
Missing data	0	3 (6%)	
**Urinary diversion**			
1. Rectosigmoid pouch			**0.030**
Yes	6 (10%)	0	
No	54 (90%)	50 (94%)	
Missing data	0	3 (6%)	
2. Colon conduit			0.50
Yes	2 (3%)	0	
No	58 (97%)	51 (96%)	
Missing data	0	2 (4%)	
3. Ileum conduit			1.0
Yes	1 (2%)	0	
No	59 (98%)	52 (98%)	
Missing data	0	1 (2%)	
**(Redo) bladder neck plasty**			0.83
Yes	15 (25%)	14 (26%)	
No	45 (75%)	38 (72%)	
Missing data	0	1 (2%)	
**Redo-ureterocystoneostomy**			0.15
Yes	8 (13%)	13 (25%)	
No	50 (83%)	38 (72%)	
Missing data	2 (3%)	2 (4%)	

Δ *Calculated by the Fisher's exact test*.

*SIS, small intestinal submucosa. Bold values are the significant ones p <0.05*.

In the cross-sectional group with a median age of 12 years, no significant differences occurred in regard to additional surgery between the two operative approaches (Table 4).

**Table 4:** Following operations for genital reconstruction or complications in the long-term follow-up.

**Operations due to complications**	**Cross-sectional patient group (*****n*** **=** **113)**		
	**Staged approach (*n* = 60)**	**Single-stage approach (*n* = 53)**	***P*^Δ^**
**Closure of urethral penile fistula**			0.64
Yes	11 (18%)	12 (23%)	
No	49 (82%)	39 (73%)	
Missing data	0	2 (4%)	
**Scar correction**			0.38
Yes	17 (28%)	10 (19%)	
No	43 (72%)	39 (73%)	
Missing data	0	4 (8%)	
Vaginal introitus plasty^*^			0.73
Yes	6 (38%)	6 (27%)	
No	10 (62%)	16 (73%)	
Missing data	0	0	
Hysterectomy^*^			1.0
Yes	0	0	
No	16 (100%)	22 (100%)	
Missing data	0	0	
Uterine sacropexy^*^			1.0
Yes	0	1 (5%)	
No	16 (100%)	21 (95%)	
Missing data	0	0	
Penile deflexion^**^			0.07
Yes	22 (58%)	13 (35%)	
No	14 (37%)	22 (60%)	
Missing data	2 (5%)	2 (5%)	

Δ *Calculated by the Fisher's exact test*.

* *Only in female patients*.

** * Only in male patients*.

Self-reported continence data were available from 20 of the 36 CBE patients reconstructed with a single-stage approach without further surgery [56%; median, 8 years (IQR, 5–10)]. Five (25%) of these were continuous continent, eight (40%) were intermittent incontinent, and three (15%) were continuous incontinent. In four patients (20%), data were missing. Four of 22 patients after a staged approach (14%); median, 10 years (IQR, 5–15) were voiding spontaneously after a bladder neck plasty as a regular part of the staged concept. None was continent; one (33%) CBE patient each reported to be intermittent incontinent and continuous incontinent. In one further patient (33%), data were missing.

## Discussion

To improve knowledge about the best care for CBE, the comprehensive EEC database of the CURE-Net was reviewed in respect of epidemiology, perioperative management, and early and long-term post-operative outcome.

Although in both sexes an increasing awareness of the high rate of inguinal hernias was observed, a considerable amount of children with CBE still do not undergo initial inguinal hernia closure at primary reconstruction. One reason might be that approximately only a quarter of hernias are clinically apparent before initial bladder closure (12). However, surgeons intraoperatively commonly find wide gaping processus vaginales; a short, steep inguinal channel; and a wide internal inguinal ring in CBE males. Additionally, one might not wish to get into an emergency situation in an EEC patient. Recent data suggested that an osteotomy might be preventive in terms of *de novo* or recurrent inguinal hernias by remodeling the anterior pelvic ring shape (13). In our cohort, neither a larger symphysis diastasis (>4 cm) (*p* = 0.45) nor the fact of having undergone osteotomy itself (*p* = 0.74) was correlated with a likelihood of an inguinal hernia. To prohibit emergency situations for inguinal hernia incarceration, concomitant inguinal herniotomy was recently recommended as a safe and effective preventive procedure during initial bladder closure (14).

Nowadays, a straightforward management with a “detailed and regimented plan” is postulated in primary and delayed closure to prevent complications and additionally an increase in costs (15–17). Although not statistically significant, prospectively observed patients were operated on more often with the staged approach at an earlier age. The reason for this decision making remains unclear. Historical considerations and experts' personal experiences might have anticipated this trend. The group from Great Ormond Street confirmed that the use of a PDC has a major impact on a successful CBE reconstruction, even being the same or more important than osteotomy (18). Considering that PDC is nowadays a standard in modern perioperative care, a rate of 59% of PDC use in the prospectively observed cohort seems to be low. Peridural catheter, however, was inserted significantly more often in the prospectively observed than in the cross-sectional cohort.

The main advantage of PDC use in Great Ormond Street for the affected babies was that intensive care unit treatment was not needed. However, more than 80% of the here analyzed German cases in both cohorts independently went postoperatively to an intensive care unit.

In this study, 36% of all CBE patients needed blood transfusions, with a significant predominance in males (*p* = 0.002). Neither age at reconstruction nor the need for osteotomy or the reconstruction technique itself did influence the necessity of blood transfusions. In the “Hopkins cohort,” blood transfusions were needed in ~58% of newborn closures (19). The authors noted no differences in terms of age and gender; however, blood transfusion was more likely with the need for osteotomy (19). This fact seems negligible in the current CURE-Net cohort, as only a vast minority had an osteotomy done. Others reported a lower transfusion rate as well; the reason for this remains unclear (20). Although patients' age at initial treatment decreased, no other fundamental changes in the perioperative management could be observed comparing the years before and after 2009, with the exception of a modest increased PDC use. Either we might conclude that treatment strategies are already optimal, or we impute that reasonable changes have sadly not been brought to daily praxis so far.

The length of urethral catheter placement after any urethral and any bladder neck reconstruction has always been a matter of debate and has a direct impact on the length of hospital stay. After primary bladder closure, the Great Ormond patients stayed in hospital significantly shorter with PDC than without (18). Seven urethral strictures occurred in the whole cohort (9.5%), leading to a bladder rupture in one case (18). Schaeffer et al. (15) reported 2% posterior bladder outlet obstruction requiring intermittent catheterization. Duration of catheter placement, however, was not reported in this series (15). Any post-operative infravesical obstruction can be disastrous for the future bladder and the upper tract development and may therefore endanger reconstruction itself (21). Although transurethral catheters remained in place significantly shorter in the prospectively observed than in the cross-sectional group, catheters in the current CURE-Net series remained longer than reported in literature before. Because of insufficient post-operative micturition, three cystoscopies were performed in the prospectively observed group (9%), detecting an anatomical stenosis in one (3%). In the cross-sectional patients, 15 cystoscopies (13%) were undertaken with eight urethral stenoses found (7%). Although there is a recent trend to earlier hospital discharge in Germany, CBE patients stay considerably longer in hospital than in other European centers (18).

Regarding the long-term outcome, complications such as upper tract dilatations seem to occur significantly more often after the single-stage procedures in the cross-sectional cohort. Urinary tract infections were generally more common after single-stage procedures in the prospectively observed and the cross-sectional cohort. Fortunately, from these data, the renal function impairment with or without the consecutive need for nephrectomy is rare in both cohorts. However, no systematic renal function assessment tests were performed in both cohorts. Urinary diversions were only reported after the staged approach. This observation is almost impossible to explain because of the retrospective data retrieval and the multicenter setting. Presumably, the operative staging is a mandatory reaction to intraoperative findings, such as a small and not compliant bladder. Therefore, it is very astonishing that long-term secondary operations, such as augmentations or catheterizable stomata, were equally distributed in the cross-sectional cohort after both primary reconstruction methods. The time periods from the initial reconstruction and the augmentations and stomata were fairly equal between the two approaches [*p* = 0.57; staged approach median, 9 years (IQR, 7–12 years); single-stage median, 9 years (IQR, 6–12 years)]. Although it must be taken in consideration that initially significantly more complications occur after single-stage reconstruction, long-term consequences are presumably the same. Additionally, a quarter of patients treated with a so-called “single-stage” approach needed further bladder neck procedures to gain continence during follow-up. This important fact confirmed by results of the CPRE concept in literature (22) should clearly be addressed during parental discussion. To gain continence is a major aim in reconstruction of the EEC. However, patients' individual outcome perspective cannot be clearly determined. In literature, continence definition and pediatric continence rates—mostly from small single-center series—vary widely from ~10 to 80%. In a systematic review on urinary continence including more than 2,500 EEC patients, only 68% of the included studies used a concise continence definition, such as “dryness with voiding or catheterization at 3-h intervals” (23). Continence according to this definition was achieved in only 51% (23). In this current evaluation, we used the current ICCS terminology (10). Comparable to a German-wide survey of 100 EEC patients aged median 13 years (IQR, 7–18 years), 33% of the patients or their families declared themselves as continuous continent, 29% described themselves as intermittent incontinent, and 28% as continuous incontinent (24). There was no significant difference in the reported continence outcome between the single-stage and the staged approach (*p* = 0.51). However, the numbers of the included patients after staged approach were substantially low. Therefore, we plan to contact all included CBE patients again to reevaluate continence status during follow-up. These independent outcome data advocate the urgent necessity to improve urinary continence status in patients with EEC at any age. A standardized definition of continence including objective and subjective criteria would be desirable.

This study has several limitations including the fact that there is no mandatory reporting of congenital malformations to a centralized birth register in Germany. Because of voluntary participation, case completeness is limited. Data retrieval was retrospective in the large amount of cross-sectional participants, so missing or inconsistent data did occur. Multicenter treatment always implies a diversity of decision making, treatment accuracy, or operative skills and disease management most probably affecting the outcome. The strengths of this study include the large multicenter and German-wide recruitment of EEC patients in general and the integrative help of the German self-help organizations and treating physicians. Personal interviews of patients and their families by specially trained physicians guarantied an extensive data collection based on clinical data and medical records.

## Conclusion

The CURE-Net data provide a trend in Germany toward the staged concept. Although prospectively observed patients were operated on at a younger age, standardized perioperative management did not significantly change in years before and after 2009. Single-stage approaches tended to have initially more post-operative complications; however, this did not result in either an increase of renal function impairment or an increase of secondary operations, such as augmentations or catheterizable stomata in the long term. Furthermore, continence outcome—although difficult to assess—did not differ between the groups and was generally lower than the reported single-center data. The results of this study suggest that following secondary operations in the long term does not differ between the different staging concepts in CBE treatment.

## Data Availability Statement

The datasets generated for this study are available on request to the corresponding author.

## Ethics Statement

This study involving human participants was reviewed and approved by each participating center's Institutional Ethics Committee (e.g., University of Regensburg No. 09/053, University of Ulm No. 425/13). Written informed consent to participate in this study was provided by the participants' legal guardian.

## Author Contributions

A-KE and NZ were the major and equally contributors in writing the manuscript. A-KE, NZ, and WR analyzed and interpreted the patient data. All authors read and approved the final manuscript. HR, EJ, RS, AH, ML, CF, FO, MF, KM, ES, MP, KH and F-MS contributed by recruiting patients and collecting data.

## Conflict of Interest

The authors declare that the research was conducted in the absence of any commercial or financial relationships that could be construed as a potential conflict of interest.
